# Correlation Between Conditional Approval and Customized Bone Implant Devices

**DOI:** 10.1111/os.12415

**Published:** 2019-03-04

**Authors:** Xiao‐lei Guo, Bin Liu, Zhong Lu

**Affiliations:** ^1^ Centre for Medical Device Evolution Beijing China

**Keywords:** Bone implant, Conditional approval, Customized device

## Abstract

This report aims to summarize key concerns regarding customized devices and conditional approval during the premarket evaluation of bone implants, and to explore the correlation between them. Based on the experience of approval of the first domestic custom‐designed bone implant, we consider the process of gaining conditional approval for urgently‐needed medical devices and medical devices for rare diseases, as well as the guidance available for clinical investigation. We also streamlined the scientifically administrative concept of this unique device, from the design and development of premarket technical evaluation to continuous post‐market study. The present study found that those two aspects have certain connections, but they are not directly correlated to each other. In contrast to the USA, Canada, Australia and the EU, where regulations and guidelines have been established for the use of customized devices, in this regard, China is still it its infancy. Thus, there is considerable potential for China to develop and perfect the policies relating to customized devices and to develop relevant strategies to ensure their efficacy with the aid of conditional approval. Appropriate scientific conditional approval for mass production of individualized anatomy‐matching bone implants could become a valuable approach for precision medicine.

## Introduction

Opinions on Deepening the Reform of the Evaluation and Approval Systems and Encouraging Innovation on Drugs and Medical Devices (File No: T. Zi [2017]42), issued by the General Office of the Communist Party of China (CPC) Central Committee and the General Office of the State Council (short as *Opinions of Two Offices* below), outlines research on paths and methods of premarket conditional approval of the medical devices used for clinically urgent drugs and rare diseases. At the same time, the Thirteenth Five‐year Plan of the Ministry of Science and Technology refers to the national key research and development programs of several customized designs related to hard tissue lesion precision medicine. In addition, there has been application, evaluation, and approval of bone implant products with customized designs in the industrial circle, and one case of a product has already been applied in the market. Thus, this report investigated the relationship between customized design and conditional approval, mainly by studying products proposed by China and other countries. We also summarize the problems to be solved and consider future possible developments.

## Classification of Approvals and Special Medical Devices

### *Regular Approval and Conditional Approval*

“Regular approval” refers to the approval of products with large universality. It does not require special conditions, because the product applied in the market is very standard, or it can be entirely or mainly approved based on former products.

In contrast, “conditional approval” is for a medical device or design that is special, with much uncertainty regarding its application in the market. It is mainly based on the “risk‐to‐benefit” ratio. Attaining conditional approval helps a device to be successfully applied. According to *Opinions of Two Offices*, an approval system should be established for medical devices, taking into consideration “clinical urgency” and “rare disease,” to reduce unforeseen risk. Thus, conditional approval should inevitably be attached for those two aspects. Certainly, there are other significant aspects of the product that require the conditional approval.

### *Special Medical Devices*

According to the US Food & Drug Administration (FDA), special medical devices can basically be divided into three types^1^, listed below.

#### *Customized Device*

A customized device, or customized design, is defined as a medical device specially designed and used for an individual patient, because the disease condition of the patient is rare, or the available products in the market cannot meet the needs of the special product and new and additional conditions must be applied. Customized devices can be further classified as “special” and “general.” Special customized bone implant devices (personalized function) tend to be for urgent clinical need or rare diseases, and require conditional approval. Generalized custom‐designed bone implants (individualized anatomy‐matching) are normally related to precision medicine for therapeutic improvement, and have conditional approval attached for their application.

The specialty of the product may comprise, but is not limited to, the product sizes, raw materials, processing methods, required mechanical and physical properties, surface properties (such as rigidity and porosity), and stress distribution. The product is specially devised by the clinician, who is the most significant person to determine the product design based on the disease. The clinician should communicate with the engineer to reach a consensus on the aspects involved. This process is called “clinician–engineer interaction.” It is obvious that the design of the final product requires the cooperation of the clinician and the engineer. Finally, because customized devices have very small sample sizes and there is a lack of statistical data, the results of the product might remain uncertain. Clinical trials should be performed from the beginning of the pre‐market study right up to the post‐market application, and products should be periodically assessed. Conditional approval should be attached to some customized device products if necessary.

#### *Patient‐matched Device*

A patient‐matched device (or patient‐specific device) refers to a device that is applied for a group of patients who have the same or similar disease conditions, and are available for clinical study. The device should be in a certain range of specification to meet the requirement of patients but differ in design from the regular medical devices. The supervision mode of patent‐matched device can be identical with that of the regular medical devices.

#### *Humanitarian Use Device*

A humanitarian use device, as noted by the FDA in 1990, is a device specially used for rare diseases. The device must comply with several rules outlined by the FDA based on the annual number of uses, risk assessment, and side effects. Table 1 presents the features of 3 special medical devices^1^.

**Table 1:** Features of three special medical devices

	Customized device	Patient‐matched device	Humanitarian‐use device
People	One patient or one clinician	One patient	Rare‐disease patients
Condition	A very special disease condition of a patient or a special operative need of a clinician	No limit	Rare disease
Limitation of use	No substitute in the USA	No limit	No substitute in the USA
Manufacturing technique	Conventional or newly‐developing technique		
Specification and size	Unique in each product against others	A range of specification and size, differing between either product	Pattern‐designed, specific specification and size, possibly identical products
Requirements of approval	Certain approval requirement of the customized device	510(k) or PMA	Certain approval requirement of the humanitarian‐use device
Post‐market surveillance	Less than 5 products annually, annual report submission to FDA	510(k) or PMA	Annual user record and report submission to IRB

FDA, Food and Drug Administration; IRB, Institutional Review Board; PMA, Premarket Approval Application.

## Features of Customized Bone Implant Devices

### *Classification*

Recent study on custom‐designed bone implants has mainly focuses on dentures or teeth^2^, ^3^, ^4^, maxillary or mandible^5^, ^6^, ^7^, and femurs^8^, ^9^, ^10^. Other notable implant types include those for the humerus, the cranium/skull, and the tibia.^11^, ^12^, ^13^, ^14^. Among all the implants introduced, it should be noted that some are applied as a substitute for bone or scaffold, while others act as additions to support or fix an original bone in the correct position. Those additions may be used, for example, for struts, framework, screws, trays, plate/template and blocks.

### *Procedure and Flow Chart*

**Figure 1:**
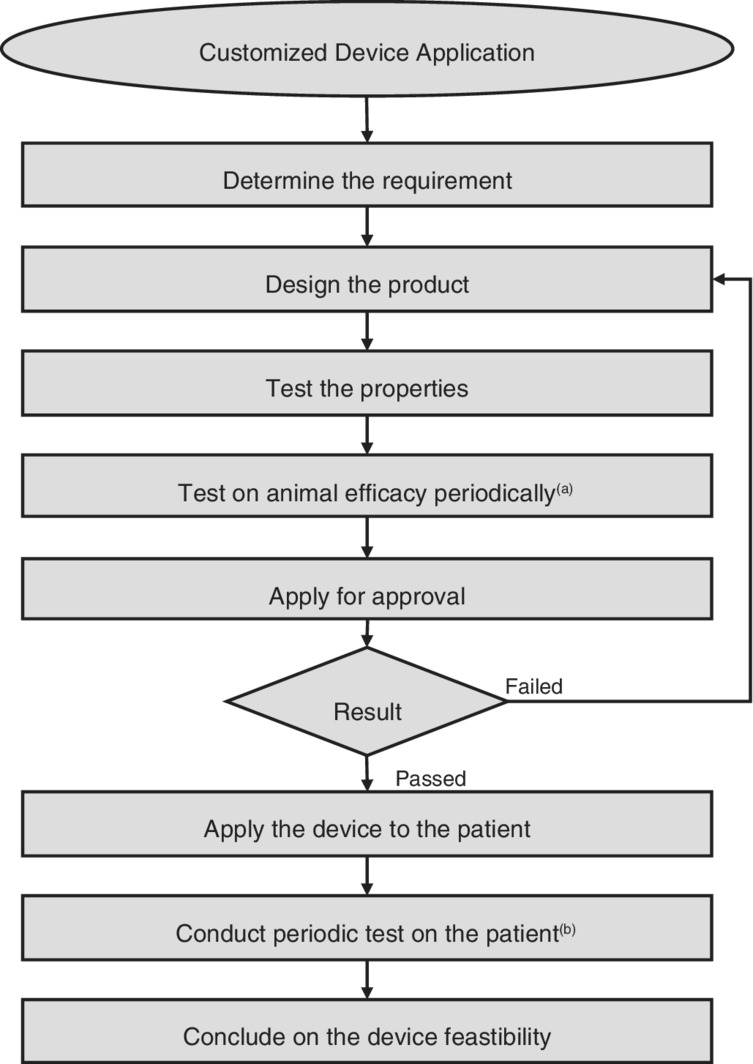
Customized device application flow chart. Notes: (a) Write periodic and detailed reports. (b) Write periodic and detailed reports to reduce risk and enhance benefits.

At first, the clinician decides whether the patient has met the requirements for a customized device. As this is determined, the clinician should discuss with an engineer the materials, properties, size and functions of the device that are suitable for the patient. Then the device is specially made. To examine whether the product can be made available for use, preclinical experiments should be conducted, examining not only biomechanical properties of the product, but also its geometry, biocompatibility, toxicity, and other necessary aspects. Animal efficacy tests are also required after those experiments. The tests should be conducted periodically and repeatedly, to analyze possibly different results and to reduce risk across different periods. The clinician and researchers should write detailed reports on the results and discussions of each test in each period. The report should contain what has occurred, what the results are, what the test indicates (positive and negative aspects), and what results are not found or require further investigation. The detailed reports support the approval of the product. When the product is approved to be used in the market, it is available for patient use. However, the clinician should also continue to conduct post‐market tests and studies. Using in‐depth studies and reports, the clinician and researchers can demonstrate whether this customized product should be made available for patient use. The flow chart of this procedure can be seen in Fig. 1.

### *Features*

The features of customized devices are summarized in what follows. Customized devices continue to have relatively large uncertainty, which mainly results from the variations between experiments. As customized devices are used relatively infrequently, there is a lack of statistical data on any one product; the sample sizes are very small. This consequently results in the unreliability of clinical tests. One possible solution is “risk assessment and classification”^15^. For statistically low‐risk products, clinical study data, retrospective data from relevant studies, and review data for congeneric products would help to reduce risk and to promote approval. For relatively high‐risk products, attention should be focused on risk assessment and clinical study reports. Conditional approval might play a significant role in the product getting approved.

Batches of experiments should be conducted before a customized device is introduced into the market. Experiments should cover, but are not limited to, size and geometry, stress distributions, and biomechanical properties (e.g. yield strength, ductility, and load‐bearing ability). The range and threshold of each quantitative value should be predetermined. In addition, the biochemical risk should be analyzed, respectively, based on “the principal stress zone” of the human motor system and “the main functional zone” of the bone implant, and division of the two zones is based on clinician–engineer interaction. The results and analyses of all those experiments are significant for the decision to approve a device.

It should be noted that the customized device has no direct relationship with conditional approval. The purpose of conditional approval is to reduce the post‐market residual risk, but a clinical study might indicate that given the risk of a customized device, the intervention of a conditional approval is necessary. When the accepted uncertainty is extremely low, conditional approval might be unnecessary. Even for a regular medical device, with large remaining risk and uncertainty, conditions should be attached with the approval.

## Features of Conditional Approval

### *Features*

The contents of a conditional approval should be based on the final ratio of risk versus benefit. When a medical device is applied in the market with conditional approval, it indicates not only that the uncertainty of the device is considerable but that expectation of benefit is larger than that of risk. It suggests that superiority of the final application is possible.

The conditional approval should pay attention to every aspect in each period of the clinical report. The obtained approval is based on multiple aspects of clinical trials and evaluations. The product standard design is the key part of the standards of a registered product.

According to *Opinions of Two Offices*, clinical urgency and rare disease are two significant aspects and customized devices derived for these purposes must be have conditional approval. “Clinical urgency” refers to not only the urgent diseases that might be life‐threatening but to urgent conditions faced during the treatment without effective solutions. “Rare disease” indicates only the uncommon diseases listed and formulated by the National Health Commission, while the “rare conditions” in the treatment of common diseases are not included in the rare diseases.

## The Worldwide Development of Customized Devices

### *Statement by the USA, Canada, Australia, and the European Union*

The regulations for customized devices are different among countries^16^.

The FDA has set limitations on the usage and management of customized devices since 2012. By 2014, Custom Device Exemption guideless were enacted, illustrating significant items of customized device applications. It states that the proposal of a customized device can be approved only when several requirements are met, including (no limited to):

1. No more than 5 products could be manufactured annually.
2. The device must be produced with the demand of a clinician with a literal report.
3. The device is applied for a special patient whose disease condition is extremely rare and no current device for this disease condition is available^17^.

The conditions for applying a customized device product in the USA are strict.

However, Canada's definition of a “custom‐made device” is slightly different from that of the USA. However, similar to the FDA of the USA, the Medical Devices Regulations of Canada (2018) claim that based on the special needs of their practice, a professional person can apply to use a “custom‐made device”. A health‐care professional should compile all necessary information regarding the patient and the device and submit this to the Ministry of Health. The Ministry has to determine the related conditions for the approval (e.g. the benefit of the device should surpass its risk, and no current device should be available in Canada)^18^. However, unlike the statement for the USA, there is no accurate limit of the annual product amount.

In 2016 in Australia, the Department of Health provided a document including information on the requirements of “custom made medical devices”^19^. It stated that this type of device is used by a health professional and incorporates a special design or special need for his/her practice. The definition did not mention its relation to patients, hence the domain of its use is further enlarged.

In a 2017 document, the European Union revised its definition of a “custom‐made device” to a device that is made “with the prescription of any person” based on his/her professional qualifications^20^. The device is used for an individual patient to meet the needs of treatment. It also outlined the detailed procedure and requirements for custom‐made device application. By comparison, the number of custom‐made devices recognized by the European Union is relatively large, and supervision and policy are loose, because the applications for devices can be approved by the related department of each country.

### *Considerations in China*

There is still no formal document from related departments in China that provides a definition for customized devices, as this is a new concept in China. However, due to the large population base of China compared to other countries, the establishment and implementation of rules for customized devices is urgently required. We should learn from the documents and practical experiences of other countries, mentioned above, and accumulate adequate knowledge and experience to set up policies for the application of customized devices in China.

## Relationship between Approvals and Devices

**Figure 2:**
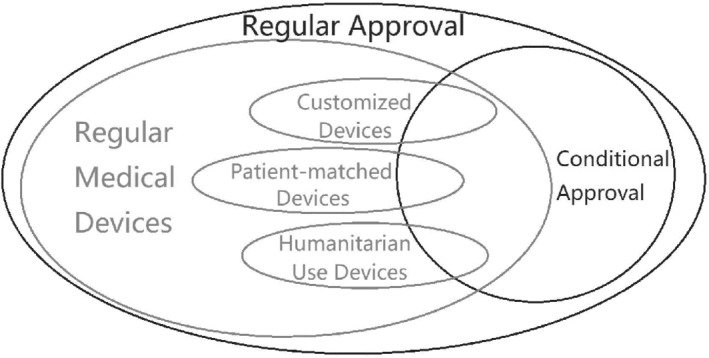
Relationship between approvals and devices.

Figure 2 illustrates the relationship between approvals and devices. Regular medical devices are separated from other special devices. As there is no limit of patients with patient‐matched devices, the sample size of this type of device is relatively larger than for customized and humanitarian‐use devices. Because conditional approval is based on risk analysis, all types of devices might receive conditional approval. Regular medical devices with conditional approval will be a significant focus of further studies.

## Conclusion

Customized devices are not directly correlated with the conditional approval, but there are some connections. Whether a customized device requires the attachment of conditional approval depends on the degree of risk and uncertainty, and whether the device is sought for clinical urgency or rare disease. Although many countries have rules and protocols for customized devices, the limitations differ from country to country. In China, this is a newly‐developed issue; hence, China should learn from other countries in implementing its own rules for customized devices.
