# Correction to “Correlation Between Conditional Approval and Customized Bone Implant Devices”

**DOI:** 10.1111/os.14074

**Published:** 2024-04-16

Guo, X.‐l. , Liu, B. and Lu, Z. (2019), Correlation Between Conditional Approval and Customized Bone Implant Devices. Orthop Surg, 11: 10–14. 10.1111/os.12415 30834707 PMC6430462

The first affiliation was erroneously published as “Centre for Medical Device Evolution, Beijing, China”. The correct affiliation should be “Center for Medical Device Evaluation, Beijing, China.”

We apologize for this error.

